# The Phytochemical Screening and Biological Properties of *Brassica napus* L. var. *napobrassica* (Rutabaga) Seeds

**DOI:** 10.3390/molecules28176250

**Published:** 2023-08-25

**Authors:** Jawaher Ayadi, Mohamed Debouba, Rami Rahmani, Jalloul Bouajila

**Affiliations:** 1 Laboratoire de Recherche, Biodiversité, Molécule et Application, Institut Supérieur de Biologie Appliquée de Médenine, Université de Gabès, Zrig, Gabès 6072, Tunisia; ayadijawaher19@gmail.com (J.A.); rahmanirami2@gmail.com (R.R.); 2 Laboratoire de Génie Chimique, Université de Toulouse, CNRS, INPT, UPS, F-31062 Toulouse, France

**Keywords:** rutabaga seeds, maceration, chemical profile, HPLC-DAD, GC-MS, in vitro bioactivity

## Abstract

Rutabaga, also known as swede and scientifically classified as *Brassica napus napobrassica*, is a biennial edible root vegetable that belongs to the *Brassica* genus and is widely cultivated in North Europe and North America. The present study highlights both the phytochemical profile and the in vitro biological properties of rutabaga seed extracts obtained through maceration using solvents of increasing polarity, namely, cyclohexane (CYHA), dichloromethane (DCM), ethyl acetate (EtOAc), methanol (MeOH), and water (H_2_O). HPLC-DAD was used to identify and quantify phenolic compounds, while volatile compounds were detected using GC-MS. The in vitro antioxidant capacity of the rutabaga seed extracts was evaluated through DPPH free radical scavenging activity. The in vitro anti-inflammatory activity (15-lipoxygenase (15-LOX) enzyme) was determined spectrophotometrically at the same concentration. Additionally, the cytotoxicity of the seed extracts was evaluated against human colon adenocarcinoma cells (Caco-2) and human embryonic kidney cells (HEK-293) using the MTT assay. The rutabaga seed extracts obtained from EtOAc, MeOH, and H_2_O were particularly rich in reducing sugars, ranging from 189.87 to 473.75 mg/g DW. The MeOH extract displayed the highest concentration of both sugars and polyphenols. Phytochemically, the HPLC-DAD analysis revealed the presence of four phenolic compounds in the tested extracts, including (±) synephrine, gallic acid, *p*-coumaric acid, and *trans*-ferulic acid, newly discovered in rutabaga organs. Moreover, a total of ten volatile compounds were identified through GC-MS analysis, both before and after derivatization. At a concentration of 50 µg/mL, the methanol extract exhibited high antioxidant activity with 52.95% inhibition, while CYHA, DCM, and EtOAc exhibited moderate anti-15-LOX activity with less than 30% inhibition. Except for DCM and aqueous extracts, rutabaga seeds did not exhibit any anti-proliferative potential against Caco-2 cell lines. Interestingly, no cytotoxicity was registered for any of the seed extracts against the normal cell line HEK-293. Overall, the obtained data highlight the potential utilization of rutabaga seeds as a source of bioactive compounds in various fields, including pharmaceuticals, nutraceuticals, and functional foods.

## 1. Introduction

Currently, special emphasis has been placed on the discovery and evaluation of new natural, effective, and non-toxic bioactive compounds with potential applications in medicinal chemistry to mitigate the various adverse effects associated with synthetic compounds in pharmaceuticals for treating various diseases [1]. Plant-derived compounds are of particular interest in this regard. They offer a vast array of chemical structures and bioactive properties that can be harnessed for therapeutic purposes [2].

The Brassicaceae family, particularly the *Brassica* (*B*) genus, has attracted substantial attention and recognition in medicinal chemistry research. This genus encompasses a diverse array of well-known species with medicinal and economic value, like turnip rape (*B. rapa*), mustard (*B. juncea*), black mustard (*B. nigra*), cabbage (*B. oleracea* var. *capitata*), broccoli (*B. oleracea* var. *italica*), cauliflower (*B. oleracea* var. *botrytis*), and the allotetraploid oilseed crop ‘*B. napus*’ [3]. Several research studies have been undertaken to analyze the phytochemical composition and investigate the biological properties of various parts of Brassicaceae family members, including roots, stems, leaves, seeds, and sprouts [3,4]. These investigations have contributed to a comprehensive understanding of the diverse chemical composition and their potential health benefits. *Brassica* species harbor an impressive repertoire of nutrients such as minerals, essential vitamins, carbohydrates, and phytochemicals, including phenolic compounds, glucosinolates and their breakdown products (isothiocyanates and indoles), carotenoids, and seed oils, recognized as bioactive secondary metabolites [5]. The metabolites synthesized by each species are responsible for providing a unique and distinct taste as well as exhibiting broadly interesting and beneficial bioactivities such as antioxidant [3], anti-inflammatory [6], antimicrobial [7], and anticancer bioactivities [8].

*B. napus*, commonly known as rapeseed (AACC, 2*n* = 38), originated from an allopolyploid between the two distinct genomes of *B. oleracea* L. (cabbage, genome CC, 2*n* = 18) and *B. rapa* (turnip, genome AA, 2*n* = 20) [9]. It is a versatile crop plant cultivated primarily for its seeds, which are valued for their high oil and protein content. In particular, rapeseed oil, which is one of the major products derived from *B. napus* seeds, holds a prominent position in the global edible oil market. It stands as the third-largest producer of edible oil worldwide, only trailing behind soybeans and palm trees [10]. *B. napus* can be classified into two distinct types: oilseed varieties, which are further categorized based on their growth habits in spring and winter, and rutabaga, scientifically identified as *B. napus napobrassica*. Rutabaga is a well-regarded agricultural crop that is particularly favored in the regions of North Europe and North America. It is frequently cultivated for its swollen edible roots, which are used in various culinary preparations, as well as for its leaves, which are consumed as leafy greens [11].

Rutabaga is a highly nutritious vegetable that contains a wide range of phytochemicals and essential nutrients. The tuber of rutabaga is particularly enriched in macronutrients such as proteins (1.20 g/100 g), fats (0.20 g/100 g), ash (0.81 g/100 g), and carbohydrates (8.13 g/100 g). Also, rutabaga is a valuable source of important minerals, including potassium (K), phosphorus (P), calcium (Ca), magnesium (Mg), and sodium (Na). Furthermore, rutabaga boasts an array of vitamins that significantly contribute to its nutritional value. It is notably rich in vitamin C, niacin (vitamin B3), pantothenic acid (vitamin B5), vitamin B6, thiamine (vitamin B1), and riboflavin (vitamin B2). In terms of fatty acids, rutabaga provides saturated fatty acids (palmitic acid and stearic acid), monounsaturated fatty acids, predominantly oleic acid, and polyunsaturated fatty acids (linoleic acid and linolenic acid) [12]. The main phenolic compounds identified in the rutabaga vegetable were apigenin, myricetin, kaempferol, and quercetin [13]. In rutabaga roots, the major glucosinolates detected were 2-hydroxy-3-butenyl glucosinolate, 4-(methylthio)butyl glucosinolate, 2-phenylethyl glucosinolate, and 3-indomethyl glucosinolate [14]. Rutabaga leaves and seeds contained aliphatic glucosinolates such as progoitrin, epiprogoitrin, sinigrin, gluconapoleiferin, glucoalyssin, gluconapin, and glucobrassicanapin. Additionally, indole glucosinolates such as glucobrassicin, 4-hydroxyglucobrassicin, neoglucobrassicin, and 4-methoxyglucobrassicin were found, along with aromatic glucosinolate gluconasturtiin [15]. Moreover, several glucosinolate-derived nitriles and isothiocyanates were detected in rutabaga cultivars [16].

This phytochemical richness attributed to rutabaga holds remarkable biological potential, particularly in terms of antioxidant and anti-proliferative activities [11]. While numerous research studies have been conducted on Brassicaceae seeds, there is limited specific scientific literature available focusing solely on the phytochemical screening and biological properties of *B. napus napobrassica* (rutabaga) seeds.

The current research study was undertaken to investigate the chemical profile of rutabaga seed extracts, which involves the quantification of total polyphenols and reducing sugars, as well as the identification of specific molecules using HPLC-DAD and GC-MS. Additionally, the study aimed to evaluate the in vitro biological proprieties, anti-DPPH, anti-inflammatory (15-LOX), and cytotoxic (Caco-2 and HEK-293) activities. A comparative analysis of their respective chemical profiles was conducted to gain a deeper understanding of the relationship between the identified molecules and the investigated biological activities.

## 2. Results and Discussion

### 2.1. Extraction Yields

Extraction serves as the crucial initial step in phytochemical analysis. In this study, the fractional extraction of rutabaga seeds has been established with maceration using various organic solvents with increasing polarity (CYHA, DCM, EtOAc, MeOH, and H_2_O). This extraction method allowed for the gradual and systematic extraction of compounds from the seeds based on their solubility properties in the distinct solvents employed.

The obtained yield values are summarized in Table 1. Regarding the extraction solvent, the highest yield percentage was recorded with cyclohexane extract (16.80%), followed by DCM one (7.50%), while the poorest was obtained with ethyl acetate (3.65%). The yields of non-polar extracts (CYHA and DCM) were higher than those of the polar extracts, indicating the presence of non-polar compounds such as fatty acids. As it is known, Brassicaceae family seeds, particularly those of rapeseed, are well recognized for their abundant oil content with high quality primarily attributed to the abundance of monounsaturated fatty acids (MUFA and oleic acid), along with significant amounts of polyunsaturated fatty acids (PUFA, omega-3, and omega-6), and low saturated fatty acid (SFA and palmitic and stearic acids) content [17].

### 2.2. Reducing Sugar Content

The quantification of reducing sugars was performed using the 3,5-dinitrosalicylic acid (DNSA) technique. As shown in Table 1, the contents of reducing sugars in different rutabaga seed extracts varied depending on the polarity of the used solvents (successively). Notably, no significant sugar content was detected in the CYHA and DCM extracts. However, the remaining extracts exhibited high sugar contents ranging from 189.87 to 473.75 mg/g DW. The highest amount was determined in the MeOH extract with 473.75 mg/g DW, accounting for 47.38% of the total initial extract (1 g). Furthermore, the EtOAc extract showed an observed sugar content of 327.40 mg/g DW, while the H_2_O extract yielded an intermediary content of 189.87 mg/g DW, representing 32.74 and 18.99%, respectively. These findings highlight the richness of rutabaga seeds in sugars and the influence of the solvent used on their extraction. Based on data from the existing literature, the reducing sugar content of rutabaga seeds surpasses that of the aqueous extract from *B. nigra* seeds, which accounted for only 5.56% of the total extract weight [18].

### 2.3. Total Polyphenol Content (TPC)

The Folin–Ciocalteu colorimetric method was used for the TPC quantification of rutabaga seed extracts. The data analysis showed a significant difference in the TPC content between extracts except for the DCM and EtOAc ones. The obtained extracts have shown a modest content of polyphenols, ranging from 3.68 to 43.77 mg gallic acid equivalents per gram of dry weight (mg GAE/g DW) (Table 1). The highest amount of TPC is reported for the methanol extract, followed by the water extract, with 43.77 and 25.23 mg GAE/g DW, respectively. However, the methanol extract obtained from the rutabaga seeds contains a substantial phenolic content, forming a minor fraction that represents only 4.38% of the total crude extract weight (1 g). This suggests the existence of a broad spectrum of different chemical components making up the remaining 95.62%, highlighting the extract’s remarkable complexity. Predominantly, the extracts contain a diverse range of chemical classes, including lipids, terpenoids, sterols, saponins, alkaloids, and glucosinolates, each possessing distinctive potential for promoting health benefits. On the other hand, the lowest content was obtained with CYHA seed extract, with only 3.68 mg GAE/g DW. Cyclohexane serves as the main solvent for lipid extraction, yielding a lipid-rich fraction characterized by an impressive extraction yield of 16.8% (Table 1). The pronounced lipid abundance appears to directly influence and contribute to the reduction in overall polyphenol levels. In comparison to data from the available literature, the present results showed a significantly higher TPC, approximately 6.3 times higher than the values reported by Pasko et al. [11] for rutabaga methanolic seed extract (6.9 mg GAE/g DW). The total phenolic content (TPC) is influenced by various factors, including the extraction methods, the polarity of the used solvent, the plant species, and the analyzed organs, as well as the stage of germination and the environmental conditions [5,19]. These factors concomitantly contribute to the overall phenolic level of the extract. Indeed, the study of Stefanucci et al. [20], conducted on the methanolic rutabaga root pulp and peel obtained with two non-conventional extraction methods, namely homogenizer-assisted extraction (HAE) and ultra-sonication-assisted extraction (UAE), revealed a variation in the TPC compared to our own results. The TPC values ranged from 10.76 to 18.14 mg GAE/g, with a higher value in the rutabaga peel UAE with 18.14 mg GAE/g. Additionally, there was a significant increase in TPC observed in the methanolic extract of the rutabaga 12-day sprouts, reaching 125.7 mg GAE/g DW, which was approximately 2.9 times greater than the amount obtained in the current study [11]. Compared to the phenolic content data reported in the review conducted by Ayadi et al. [4] for various *Brassica* species seeds, our findings reveal significantly elevated levels, specifically when compared to the previous studies focused on the seeds of *B. oleracea* [21], *B. hirta* [22], and *B. juncea* [23]. However, it is important to note that the phenolic content in canola (*B. napus*) seeds and Dolsan mustard seeds (*B. juncea*), with, respectively 462.3 μg/mg and 404.33 mg GAE/g extract, still surpasses the levels observed in our study [24,25].

### 2.4. The Identification and Quantification of Phenolic Compounds Using HPLC-DAD

**Figure 1:**
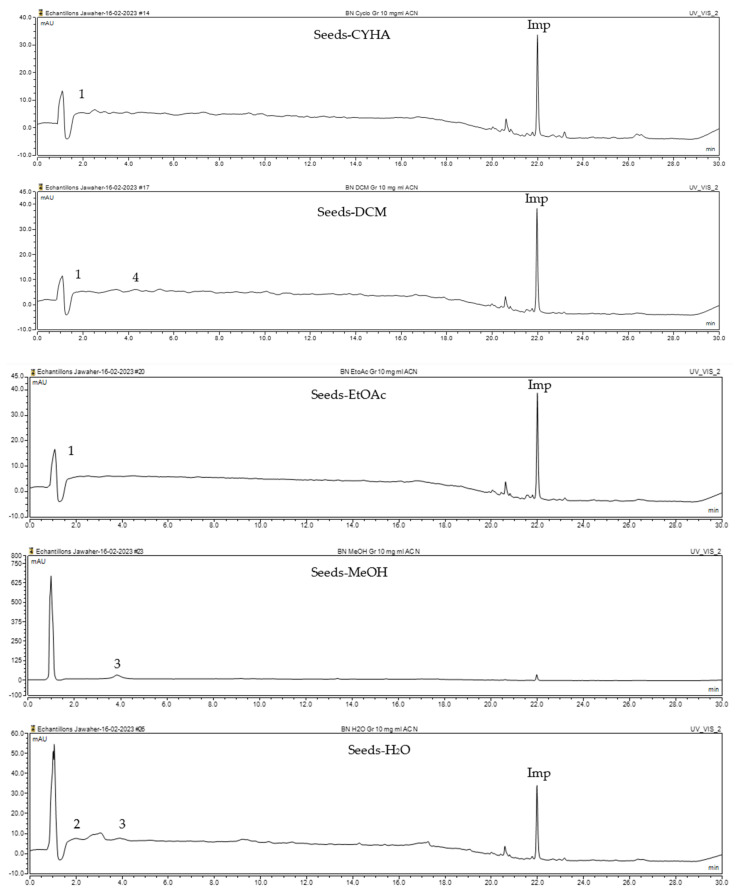
HPLC-DAD chromatograms at 280 nm of rutabaga seed extracts (CYHA: cyclohexane, DCM: dichloromethane, EtOAc: ethyl acetate, MeOH: methanol, water: H_2_O): (±) synephrine (1), gallic acid (2), *p*-coumaric acid (3), and *trans*-ferulic acid (4). Imp: impurity of mobile phase.

The HPLC-DAD method was employed to identify and quantify phenolic compounds in various rutabaga seed extracts. In total, only four compounds have been successfully identified by comparing the retention time and λmax of each peak with those of the ninety-four standards injected under precisely identical conditions as the extracts, including (±) synephrine (y = 0.385x + 0.5679), gallic acid (y = 0.9282x − 4.35), *p*-coumaric acid (y = 4.8392x + 4.714), and *trans*-ferulic acid (y = 2.1952x + 14.822) (Table 2). This comparison enabled a precise determination and confirmation of the presence of these compounds in the samples. The quantification of each compound was expressed as milligrams per gram of the respective total extract (Table 2). All the identified phenolic compounds were found for the first time in rutabaga seeds, whereas gallic acid, *p*-coumaric acid, and *trans*-ferulic acid were previously detected in Brassicaceae family organs. Some compounds were found to be present in two or three extracts. Indeed, (±) synephrine was detected in CYHA, DCM, and EtOAc extracts at 0.21, 0.27, and 0.25 mg/g extract, respectively. Also, *p*-coumaric acid was identified in the two polar extracts, MeOH and H_2_O. Interestingly, out of all the detected compounds, *p*-coumaric acid showed the highest concentration compared to the other identified compounds, followed by *trans*-ferulic acid. The MeOH extract was particularly rich in *p*-coumaric acid, with an impressive concentration of 5.64 mg/g extract, while the DCM extract stood out for its notable content of *trans*-ferulic acid, reaching 1.62 mg/g extract. Based on the available literature, there has been a lack of research focused on the chemical profile of rutabaga seeds. In the previous study by Pasko et al. [26], the qualitative and quantitative HPLC analyses revealed the presence of several phenolic acids and flavonoids in the rutabaga seeds’ methanol extract that were distinctly not identified in the current study. Notably, significant quantities of caffeic acid (3.75 mg/g DW), sinapic acid (1.50 mg/g DW), and chlorogenic acid (0.44 mg/g DW) were accompanied by minimal amounts of ferulic acid. In the same study, robinin and isoquercitrin were found to be the major flavonoids with 2.18 and 0.55 mg/g DW, respectively, while quercetin, myricetin, Kaempferol, morin, and apigenin were also identified in trace amounts. In comparison to data from the available literature on the chemical profile of the Brassicaceae family, gallic acid, found in aqueous extract, was previously detected in *Eruca sativa mill*. defatted seed meal extract by Testai et al. [27] with a concentration of 1.45 mg/g. Additionally, *p*-coumaric acid, the highest compound, was quantified in the *B. alba*, B. *juncea*, and *B. nigra* seeds with 0.0005, 0.0002, and 0.0001 mg/g, respectively [28]. Furthermore, *trans*-ferulic acid, the second-highest compound identified in the DCM extract, was also detected in various extracts of *B. oleracea* L. var. *Sabellica* obtained using ultrasound-assisted extraction, with concentrations ranging from 0.002 to 0.003 mg/g of DW [29]. These findings highlight that the content of those compounds is significantly higher in our study compared to the data previously reported in the literature (Figure 1).

### 2.5. The Identification of Volatile Compounds in Rutabaga Seed Extracts Using GC-MS (before and after Derivatization)

The GC-MS analysis was conducted to examine the composition of volatile compounds present in different rutabaga seed extracts. This analytical technique allowed for the identification of individual compounds within the seed extracts based on their mass spectra. In an attempt to identify additional volatile compounds within the various extracts, a derivatization reaction was employed. This reaction aimed to generate silylated products with enhanced chromatographic properties and volatility, facilitating their separation and identification. The GC-MS data analysis revealed the presence of a total of ten compounds, both before and after derivatization, classified as polyphenols (including phenol, 3,5-bis(1,1-dimethylethyl)- and 2,4-Di-tert-butylphenol), fatty acids derivatives (such as tetracosanoic acid, methyl ester, and docosanoic acid), organic acid (carbonic acid), two alcohols (including ethylene glycol and glycerol), and monosaccharide (methyl α-D-glucofuranoside) (Table 3). It is important to highlight that the identified volatile compounds were newly discovered in rutabaga whole parts and precisely in seeds. Some compounds detected in the rutabaga seed extract of this study had previously been identified and reported in various organs belonging to the Brassicaceae family. For instance, 2,4-Di-tert-butylphenol, detected in EtOAc extract before derivatization, was found in *B. oleracea* L. seeds [31]. Furthermore, glycerol, detected in DCM extract after derivatization, was also identified in the leaves of six edible cruciferous vegetables, including cauliflower, turnip, broccoli, watercress, radish, and cabbage. Notably, broccoli exhibited a high level (5.0%), while cabbage demonstrated a comparatively low level (0.5%) [32].

**Figure 2:**
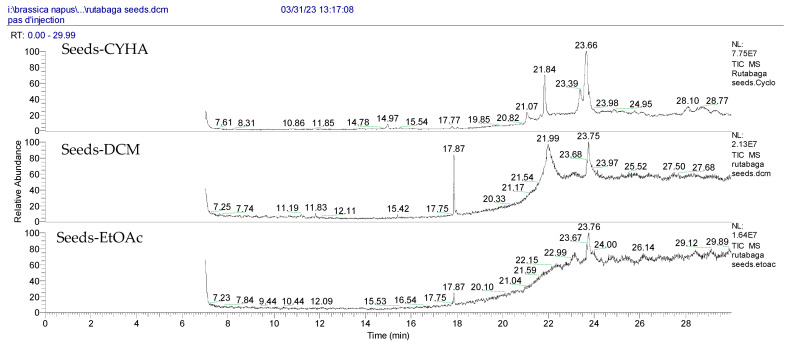
GC-MS chromatograms of rutabaga seed extracts (CYHA: cyclohexane, DCM: dichloromethane, EtOAc: ethyl acetate).

Notably, a total of seven compounds, representing 70% of the detected molecules, were successfully identified, both before and after derivatization, in non-polar extracts (CYHA and DCM) (Figure 2), highlighting their non-polarity and indicating their affinity for non-polar solvents during extraction. Docosanoic acid, methyl ester was found to be the dominant compound detected in the CYHA extract before derivatization, making up 11.13% of the extract composition. This was followed by tetracosanoic acid, methyl ester and 1,1’-bicyclohexyl, with area percentage values of 4.64% and 1.46%, respectively. Within the DCM extract, only phenol, 3,5-bis(1,1-dimethylethyl) was identified, accounting for 6.60% of the extract’s composition, while the EtOAc extract revealed the exclusive presence of only 2,4-Di-tert-butylphenol, constituting 1.77% of the extract’s content. Conversely, no volatile compounds were detected either without or with derivatization in the methanol extract. The notable differences observed in the volatile profile and peak areas among various solvents indicate variations in the solubility of compounds and their distinctive affinities for each solvent, as well as their volatility, a crucial factor influencing their detectability through GC-MS analysis. According to the existing literature, the available data regarding the volatile profile of rutabaga seeds remains limited. The GC-MS method was employed to investigate the fatty acid profile. Specifically, an investigation conducted on the fatty acid analysis of rutabaga seed methanol extract revealed the predominance of unsaturated fatty acids with low levels of saturated fatty acids. The main unsaturated fatty acids identified were erucic acid (22:1) and linoleic acid (18:2), accounting for 42.5% and 17.5% of the composition, respectively. Stearic acid (18:0) was the major saturated fatty acid, comprising around 4.2% of the total fatty acids. Minor amounts of capric acid (10:0) and palmitic acid (16:0) were also detected [26]. Our seeds have a low content of fatty acids.

### 2.6. Antioxidant Activity with DPPH Assay

**Figure 3:**
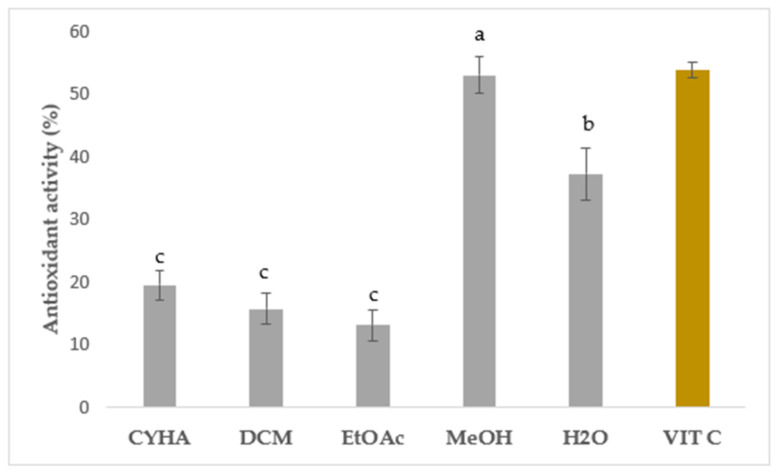
Antioxidant assay of rutabaga seed extracts at 50 µg/mL and vitamin C at 4 µg/mL. Results are mean ± SD (*n* = 3). The presence of distinct letters on the histograms indicates a significant difference between extracts according to Tukey’s test (significance level of *p* ≤ 0.05).

In this study, the DPPH free radical scavenging activity was measured to assess the in vitro antioxidant capacity of rutabaga seed extracts tested at 50 µg/mL and compared with the standard vitamin C (IC_50_ = 4 μg/mL). Results were expressed as inhibition percentage (%) and are illustrated in Figure 3. Statistically, a significant difference was seen between the extracts. Regarding the extraction solvents, the moderate antioxidant activity was ranked from 13.12 (EtOAc) to 52.95% (MeOH), with a higher quenching capacity in the polar extracts than the apolar ones. Similar to the total phenolics, the methanolic extract exhibited the most potent DPPH free radical scavenging activity with 52.95%. Aqueous extract showed the second-most effective extract with moderate inhibition (37.20%). Concerning the non-polar solvents, a weak ability to neutralize DPPH free radicals was registered with CYHA (19.43%) and DCM (15.72%) extracts. The EtOAc extract presented the lowest inhibition with only 13.12%. These findings suggest that the polar molecules seem to be primarily responsible for the free radical scavenging capacity. The observed variation in inhibition activity can be attributed to the presence of a potential correlation between the specific chemical composition of each extract and its respective inhibitory effects. The significant quenching activity observed in the MeOH and aqueous extracts can be attributed to the remarkable antioxidant capability of *p*-coumaric acid [41] and gallic acid [42], along with other essential molecules like vitamins, carotenoids, glucosinolates, and triterpenes. The synergistic interaction between different phytochemicals enhances the overall therapeutic potential and health benefits derived from *Brassica* plants [43]. The assessment of in vitro antioxidant activity has garnered significant attention in various studies focusing on the seeds of different *Brassica* species. Numerous reports have emphasized the robust antioxidant potential exhibited by these seeds, particularly in terms of their notable DPPH radical-scavenging activity. Drawing upon the existing data, our current study demonstrated the superior effectiveness of the rutabaga seed methanolic extract with an IC_50_ of 50 µg/mL in comparison to the alcoholic extract (IC_50_ = 103 µg/mL), the ethanolic extract (IC_50_ = 170 µg/mL), and the aqueous extract (IC_50_ = 390 µg/mL) of *B. juncea* [44,45].

### 2.7. Biological Activities

#### 2.7.1. Anti-Inflammatory Activity

The in vitro rutabaga seed extract’s ability to inhibit the 15-lypoxygenase (15-LOX) enzyme was determined spectrophotometrically at 50 µg/mL and Nordihydroguaiaretic acid (NDGA) at 4 µg/mL was used as a positive reference. The obtained results are represented in Figure S1, Supplementary Materials. The EtOAc, CYHA, and DCM seed extracts showed a moderate anti-15-LOX potential effect that did not exceed 30%. Conversely, no inhibition was registered for MeOH and H_2_O extracts. The findings obtained in this study fell within the range of previously reported results by Rahmani et al. [46] in their study on *B. tournefortii* leaves (13–19%), stems (16–24%), and roots (8–28%). The observed behavior against the 15-LOX enzyme can be attributed to the presence of molecules such as *trans*-ferulic acid, detected in DCM extract, and 2,4-Di-tert-butylphenol, detected in EtOAc extract, which were strongly associated with the anti-inflammatory effect [47,48,49]. Furthermore, it is also important to highlight that, despite its low TPC, the CYHA extract displayed the highest 15-LOX inhibition percentage. This suggests that the bioactivity of the extracts may be associated with other chemical classes of metabolites, such as fatty acids, in addition to phenolic compounds [50].

#### 2.7.2. Cytotoxic Activity

The MTT assay was employed to assess the anti-proliferative effect of the obtained extracts of rutabaga seeds on human colon adenocarcinoma cells (Caco-2) and to quantify their cytotoxicity in human embryonic kidney cells (Hek-293) after treatment for 48 h. Tamoxifen was used as a reference at 100 µM. Concerning the Caco-2 cells, as shown in Figure S2, Supplementary Materials, the highest growth inhibition of Caco-2 was recorded with the DCM extract (27.93%), followed by H_2_O (5.34%). No inhibition was registered with the CYHA, EtOAc, and MeOH extracts. An investigation conducted by Miceli et al. [51] examined the anti-proliferative impact of the leaf and flowering top extracts of *B. incana* at various concentrations (ranging from 0.0625 to 2 mg/mL) against Caco-2 and revealed that the highest concentration of flowering top extract (2 mg/mL) was the most potent, exhibiting inhibitory effects of approximately 90% with IC_50_ = 1.25 mg/mL and 1.1 mg/mL at 48 h and 72 h, respectively. The moderate inhibition of Caco-2 cell proliferation with the DCM extract can be partly attributed to the presence of some compounds known for their anticancer effects, such as phenol, 3,5-bis(1,1-dimethylethyl)-, and glycerol detected using GC-MS [52,53]. Additionally, *trans*-ferulic acid, identified through HPLC-DAD, exhibited cytotoxic activity against Caco-2 cells, with an IC_50_ value of 12.58 µM [47].

**Figure 4:**
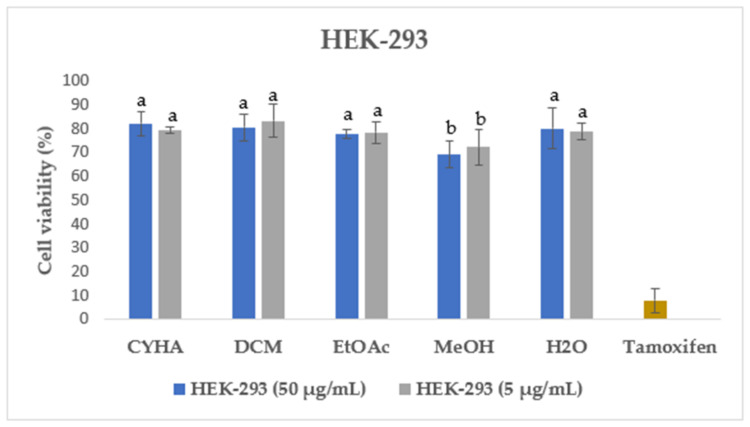
Cell viability of HEK-293 after 48 h treatment with 50 µg/mL and 5 µg/mL of rutabaga seed extract. Tamoxifen was tested at 100 µM. Results are mean ± SD (*n* = 3). The histograms’ different letters indicate significant difference according to Tukey’s test (*p* ≤ 0.05).

Additionally, the cytotoxicity of different rutabaga seed extracts was also assessed with the MTT assay in normal HEK-293 cells at two concentrations of 50 µg/mL and 5 µg/mL. HEK-293 cells were originally derived from human embryonic kidney cells transformed with sheared adenovirus type 5 DNA fragments [54]. They are considered immortalized cells with the ability to undergo indefinite cell division, which makes them valuable and widely used in biomedical research [55]. As shown in Figure 4, for all the extracts tested both at 50 µg/mL and 5 µg/mL, a high percentage of HEK-293 cell viability, exceeding 69%, was recorded. In the same context, the previous study conducted by Malfa et al. [56] revealed that the leaves crude extract of *B. Villosa* subsp. *Drepanensis*, at all tested concentrations (25–1000 μg/mL), exhibited no impact on healthy normal cell (HFF-1 and RAW 264.7) viability. Those findings highlight the potential safety and non-toxic nature of the rutabaga extracts, suggesting their suitability for further exploration and potential applications in various fields such as pharmaceuticals, nutraceuticals, or functional foods.

### 2.8. Principal Component Analysis (PCA)

In this study, the principal component analysis (PCA) (Table 4) was utilized to establish the correlation between the total phenolic content (TPC) and the various assessed biological properties of the rutabaga seed extracts. The PCA analysis did not take into consideration the reducing sugar content due to the lack of significant biological proprieties of sugars concerning the investigated biological activities. The axes of inertia have been concealed from this analysis.

**Figure 5:**
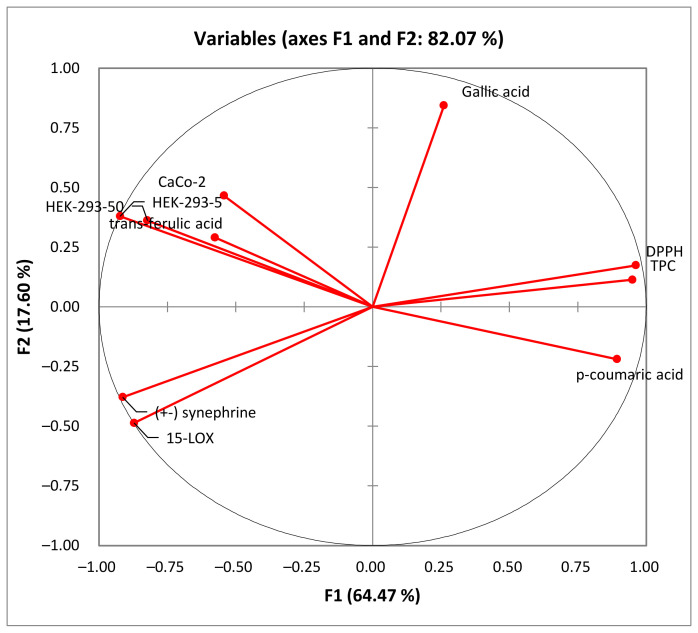
Principal component analysis “loading plot” of the total phenolic content (TPC), the antioxidant activity (DPPH) and biological activities (anti-inflammatory activity: 15-LOX), and the cytotoxic activity (Caco-2 and HEK-293) of rutabaga seed extracts.

As shown in Figure 5, the percentage of total variation was recorded at 82.07%. PC1 and PC2 axes accounted for 64.47% and 17.60% of the variability, respectively.

In the PCA loading plot, the loadings reflect both the correlation of the principal components with the original variables and the correlations between the different activities and total phenolic content and the identified phenolic compounds. Based on the information provided in Table 5, several correlations can be observed. PC1 was strongly correlated with TPC, DPPH, and *p*-coumaric acid, with 0.950, 0.963, and 0.893, respectively. Conversely, PC1 exhibited negative correlations with the level of anti-inflammatory activity (15-LOX) (r = −0.872), cytotoxic activity against HEK-293 cells (r = −0.823 and r = −0.923) and Caco-2 cells (r = −0.543), (±) synephrine (r = −0.914), and *trans*-ferulic acid (r = −0.577). Additionally, PC2 was well correlated with gallic acid, with a loading of 0.844. For the PC3, there was a good positive correlation with the cytotoxicity against Caco-2 cells and *trans*-ferulic acid with loadings of 0.699 and 0.764, respectively.

**Figure 6:**
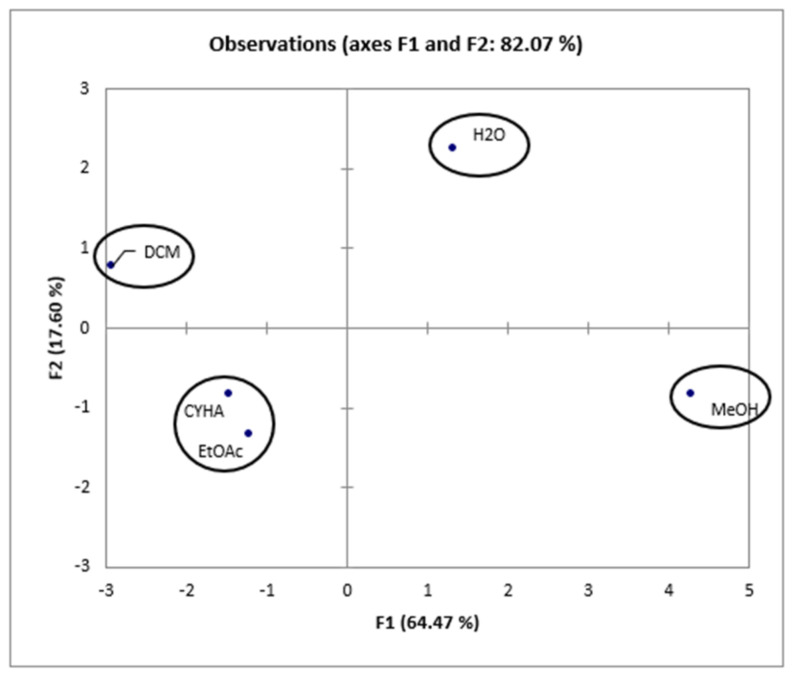
Principal components analysis “score plot” of rutabaga seed extracts (CYHA: cyclohexane, DCM: dichloromethane, EtOAc: ethyl acetate, MeOH: methanol, water: H_2_O).

Taking into account the information presented in the correlation matrix (Table 6), a strong correlation was established between the total phenolic content and the DPPH activity (0.958), proving the effectiveness of phenolic compounds in scavenging DPPH free radicals. Conversely, TPC is negatively correlated with 15-LOX (r = −0897), HEK-293 (r = −0.867, r = −0.820), and Caco-2 (r = −0.271) activities, which suggests the presence of other bioactive compounds in the extracts, such as fatty acids. Additionally, 15-LOX presents a moderate correlation with cytotoxic activity in HEK-293 (0.559 and 0.616) and a low correlation with cytotoxic activity against Caco-2 (0.208). Furthermore, for the cytotoxic activity, a high correlation (0.718) was presented between HEK-293 (5 µg/mL) and Caco-2, which suggests the non-toxicity of low-concentration rutabaga seed extracts against both normal and cancer cell lines. However, at a concentration of 50µg/mL of rutabaga seed extracts, a low correlation (0.342) was established between the cytotoxic activity of the two cell lines. This could be explained by the moderate inhibition of extracts against Caco-2 and their safety in the normal cell line HEK-293. Furthermore, *trans*-ferulic acid was probably the most efficient molecule against Caco-2 cells, with a strong positive correlation (r = 0.982). Based on Figure 6, the different extracts can be classified into four groups: C1 (MeOH), C2 (H_2_O), C3 (CYHA and EtOAc), and C4 (DCM).

**Figure 7:**
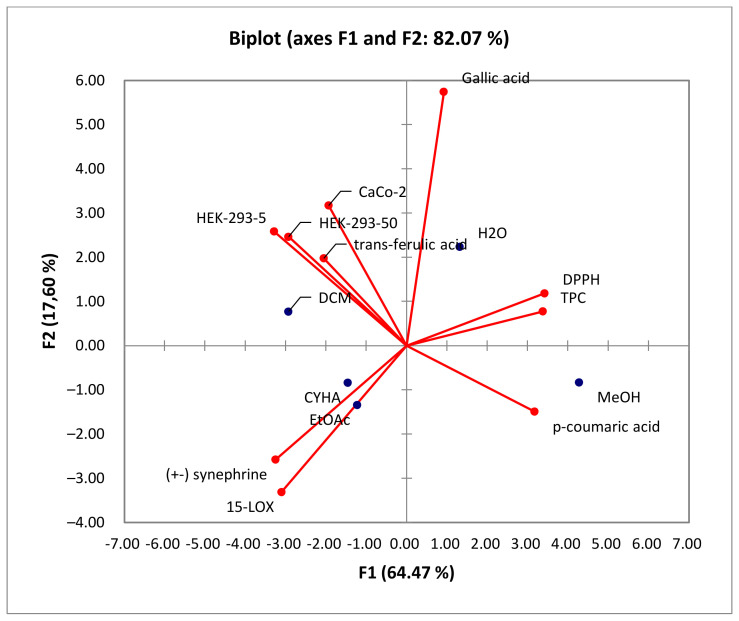
Principal components analysis “Biplot” of the total phenolic content (TPC), the antioxidant activity (DPPH), biological activities (anti-inflammatory activity: 15-LOX), and cytotoxic activity (Caco-2 and HEK-293) of rutabaga seed extracts.

The correlation between variables and observations is illustrated in Figure 7. Based on the Biplot figure, it appears that the extracts’ placement in relation to TPC and biological activities was influenced by their specific chemical composition. The results demonstrated that the high anti-DPPH activity of the methanol extract can be attributed to its richness in TPC, particularly in *p*-coumaric acid (r = 0.889) [41]. This finding suggests a strong correlation between the presence of phenolic compounds and antioxidant activity. Furthermore, the 15-LOX activity was associated with the CYHA and EtOAc extracts, indicating the presence of other compounds like fatty acids, known according to the literature by their anti-inflammatory activity [50]. The cytotoxic activity in HEK-293 was found to be linked to the DCM extract. This indicates that the moderate anti-inflammatory and cytotoxic activity observed in the study are not directly correlated with the presence of phenolic compounds. The richness of DCM seed extract with *trans*-ferulic acid, along with other molecules such as phenol, 3,5-bis(1,1-dimethylethyl)-, and glycerol detected using GC-MS, could explain its cytotoxic activity against Caco-2 cells [47,52,53].

## 3. Materials and Methods

### 3.1. Plant Material and Reagents

The rutabaga seeds used in this study were provided by Botanic Company (France). The seeds were planted and nurtured during the period of May to July and harvested from September to December. The seeds were crushed with a pestle and mortar to increase their surface area and facilitate the extraction process.

Cyclohexane (CYHA), dichloromethane (DCM), ethyl acetate (EtOAc), methanol (MeOH), acetonitrile (ACN), acetic acid, N, O-bis(trimethylsilyl)trifluoroacetamide (BSTFA), dimethyl sulfoxide (DMSO), 3,5-dinitrosalicylic acid (DNSA), sodium potassium tartrate, sodium hydroxide (NaOH), Folin–Ciocalteu reagent (2 N), sodium carbonate, 1,1-diphenyl-2-picrylhydrazyl (DPPH), ascorbic acid, potassium dihydrogen phosphate (KH_2_PO_4_), sodium dihydrogen phosphate (Na_2_HPO_4_), nordihydroguaiaretic acid (NDGA), 15-Lypoxygenase enzyme (15-LOX), Dulbecco’s modified eagle medium (DMEM), foetal bovine serum (FBS), non-essential amino acids, penicillin, streptomycin, gentamicin, thiazolyl blue tetrazolium bromide (MTT), and analytical standards were purchased from Sigma-Aldrich (France). The analytical standards used to identify and quantify the main phenolic compounds present in the seed extracts were: trihydroxyethylrutin; rutin; catechin; 3,4-dihydroxy-5-methoxybenzoic acid; quercetin 3-β-D-glucoside; polydatin; 2,4-dihydroxycinnamic acid; ellagic acid; (±) synephrine; chlorogenic acid; gallic acid; (−)-epicatechin; gallocyanine; brilliant yellow; methyl 3,5-dihydroxybenzoate; 3-amino-4-hydroxybenzoic acid; 3,4-dihydroxycinnamic acid (caffeic Acid); sinapic acid; *trans*-3-hydroxycinnamic acid; *p*-coumaric acid; *trans*-ferulic acid; 4,7-dihydroxycoumarin; 7-hydroxycoumarin-3-carboxylic acid N-succinimidyl ester; 7-hydroxycoumarin-3-carboxylic acid; methyl 4-hydroxybenzoate; myricetin; 6-hydroxycoumarin; coumarin; 7-hydroxy-4-methyl-3-coumarinylacetic acid; 3-cyanoumbelliferone; isopropyl 3,4,5-trihydroxybenzoate; resveratrol; 4-hydroxy-3-methoxycinnamic acid; 2-hydroxycinnamic acid; quercetin; ethyl 3,4-dihydroxycinnamate; 7,8-dihydroxy-2,2-dimethylchromane-6-carboxylic acid; *trans*-cinnamic acid; 3,4-dihydroxy-benzoic acid methyl ester; 4-ethyl-7-hydroxy-8-methyl-2H-chromen-2-one; α-cyano-4-hydroxycinnamic acid; naringenin; trolox; (±)-taxifolin; 7,3′-dihydroxyflavone; 2,4-dihydroxy-3,6-dimethylbenzoic acid; 3-cyano-7-hydroxy-4-methylcoumarin; (±)-6-hydroxy-2,5,7,8-tetramethylchroumane-2-carboxylic acid; butyl gallate; 6-hydroxyflavone; baicalein; ethyl 3,5-dihydroxybenzoate; ethyl *trans*-2-hydroxycinnamate; kaempferol; 5,8-dihydroxy-1,4-naphthoquinone; ethyl 4-hydroxy-3-cinamate; 7-hydroxy-4-phenylcoumarin; 2-chloro-3-(4-hydroxy-phenylamino)-(1,4) naphthoquinone; 3-chloro-7-hydroxy-4-methylcoumarin; 5-hydroxy-4′-methoxylflavone; chrysin; warfarin (4-hydroxy-3-(3-oxo-1-phenylbutyl)coumarin); icariin; 3′-hydroxy-a-naphthoflavone; 3-tert-butyl-4-hydroxybenzoic acid; 5,7-dihydroxy-3′,4′,5′-trimethoxyflavone; 7-hydroxyflavone; *β*-carotene; lutein; 4-hydroxytamoxifen; 5,7-dihydroxy-4-propylcoumarin; shikonin; 3′-hydroxy-6-methylflavone; 7-hydroxy-4-(trifluoromethyl) coumarin; 5-hydroxyflavone; isobutyl 4-hydroxybenzoate; 3,3′,4′_trimethoxyflavone; diethylstilbestrol; butyl 4-hydroxybenzoate; 7-hydroxy-3′,4′,5′-trimethoxy-alpha-naphthoflavone; 3′-hydroxy-b-naphtoflavone; 3,3′-dimethoxyflavone; 2,3-dichloro-5,8-dihydroxy-1,4-naphthoquinone; 3,6,3′-trimethoxyflavone; 3,7-dimethoxyflavone; 5-hydroxy-3′-methoxyflavone; xanthurenic acid; 4′,5′-dimethoxy-2′-hydroxy-4-methylchalcone; rosmarinic acid; (z)-3-(3-ethoxy-4-hydroxy-phenyl)-2-phenyl-acrylic acid; 2-chloro-3-(3,5-di-tert-butyl-4-hydroxy-phenyl)(1-4)naphtoquinone; hamamelitannin; 9-chloro-10-hydroxy-2,3-diméthyl-anthracène-1,4-dione; 3,4-dihydroxy-5-methoxycinnamic acid; and 5-hydroxy-7-((3-methylbenzyl)oxy)-2-phenyl-4h-chromen-4-one.

### 3.2. Extraction

The maceration method was used to extract the secondary metabolites from rutabaga seeds. Twenty grams of rutabaga seed were extracted continuously for 2 h with moderate agitation with increasing-polarity organic solvents: CYHA, DCM, EtOAc, MeOH, and H_2_O (sample to solvent ratio of 1:10 (*w*/*v*)) at room pressure and temperature. Filtered extracts were concentrated via distillation in a rotary evaporator under vacuum with reduced pressure at 35 °C (IKA, RV 10 auto V, Germany). The following formula was used to determine the extraction yield:

Yield (%) = (m/M) × 100,

with m: weight of dry weight (g); M: weight of plant material (g).

### 3.3. Quantification of Reducing Sugar Content

The reducing sugar quantification (RSC) of the rutabaga seed extracts was determined using the 3,5-dinitrosalicylic acid (DNSA) method as described by Kouhoude et al. [57] with slight modifications. Briefly, 150 µL of each extract (350 mg/L) was combined with 150 µL DNS solution. Following a 5 min incubation at 100 °C with stirring, 750 μL of deionized water was introduced. Subsequently, after a second round of stirring, the mixture’s absorbance was measured at 530 nm in comparison to a reference blank consisting of DNS solvent (sodium potassium tartrate in NaOH (2M)) instead of DNS, and to negative control where the extract was substituted with DMSO. The sugar content was determined in milligrams of glucose equivalent per gram of dry residue (mg GAE/g DW).

### 3.4. Total Phenolic Content (TPC)

The total phenolic content of different obtained extracts was estimated with a colorimetric assay using the Folin–Ciocalteu method as described by Ben Khadher et al. [58]. In a basic environment generated by the sodium carbonate (Na_2_CO_3_) and upon oxidation of the sample’s phenols, the Folin–Ciocalteu reagent’s phosphotungstic acid and phosphomolybdic acid were reduced to a blue-colored complex, which was proportional to the amount of phenolic compounds present. The blue color intensity was assessed with a microplate reader (Multiskan Go, F1-01620, Thermo Fisher Scientific, Vantaa, Finland) at 765 nm. TPC content was reported as milligrams of gallic acid equivalents per gram of dry weight (mg GAE/g DW) using the regression equation derived from the standard calibration curve of known gallic acid concentrations (0 to 115 mg/L) (R^2^ = 0.996).

### 3.5. High-Performance Liquid Chromatography Analysis (HPLC-DAD)

For the chromatographic analysis, the dried extracts were redissolved in acetonitrile with a concentration of 10 mg/mL and filtered through 0.2 µm polytetrafluoroethylene (PTFE) membrane filters. Twenty microliters of the prepared samples were then subjected to the Thermos Scientific Spectra Accela pump coupled to the PDA detector Waters 996 (the detection wavelength was 280 nm). The separation of compounds was performed through an RP-C18 reversed-phase column of 25 cm × 4.6 mm and 5 µm particle size (Phenomenex, Le Pecq, France), thermostated at 25 °C. The samples were eluted into the column through the mobile phase consisting of a mixture of acidified water (pH = 2.65) (solvent A) and acidified water/ACN (20:80 *v*/*v*) (solvent B). Elution was carried out at a constant flow rate of 0.5 mL/min with the following linear gradient of increasing solvent B concentration starting from 12% B and reaching 30% B for 15 min, followed by a 2 min transition from 30% B to 50% B, another 3 min transition from 50% B to 99.9% B, and finally a 3 min equilibration phase from 99.9% B back to 12% B. The identification of phenolic compounds was achieved by comparing the retention time and λmax values with those of established standards. Furthermore, the quantification of each identified compound was performed through their specific calibration curves.

### 3.6. Gas Chromatography Mass Spectrometry (GC-MS) Analysis

The volatile profile screening of the rutabaga seed extracts, before and after derivatization, was conducted using the method described by Ben Khadher et al. [58] with slight modifications. For analysis purposes, the samples were prepared with a concentration of 5 mg/mL in their principal extraction solvents except for methanol and water extracts, where acetonitrile was used. GC-MS analyses were performed on a Trace 1300 Gas Chromatograph (France) equipped with a DB-5MS column and coupled to triple quadrupole mass-spectrometer (TSQ 8000 Evo). H_2_ was the carrier gas. The column oven temperature program was as follows: 60 °C held for 1 min, followed by a gradual increase in temperature at a rate of 10 °C/min to reach 150 °C, then 1 min isothermally at 150 °C. Thereafter, another gradient was applied to reach 260 °C at 12 °C/min, then held for 10 min. The mass spectrum of each acquisition was registered in full-scan mode from 70 to 800 AMU with an ion source held at 220 °C and a transfer line heated at 240 °C. Five microliters of each extract were injected. The identification of compounds in the extracts was completed via the comparison between their mass spectra and those proposed by the NIST08 database (National Institute of Standards and Technology, https://www.nist.gov/, MS library version 2.4 build 25 March 2020).

Derivatization method:

The derivatization method consisted of adding to 290 µL of prepared samples, as previously reported, 60 µL of N, O-bis(trimethylsilyl)trifluoroacetamide (BSTFA) reagent and incubating the reaction mixture at 40 °C for 30 min [58]. The spectra analysis of every derivative solution was performed as described in the previous section.

### 3.7. Free Radical Scavenging Activity DPPH Test

The free-radical-scavenging capacities of the obtained extracts were determined with a spectrophotometric assay using the stable radical DPPH• (1,1-diphenyl-2-picrylhydrazyl) developed by Ben Khadher et al. [58]. In a 96-well microplate (Micro Well, Thermo Fisher Scientific, Illkirch, France), a total volume of 200 µL was prepared by adding 20 µL of each diluted rutabaga seed extract (0.5 mg/mL) to 180 μL of 0.2 mM methanolic DPPH solution. Ascorbic acid, at a concentration of 4 µg/mL, was used as the reference compound. After a 25 min period of incubation at 25 °C in darkness, the absorbance was measured at 524 nm with a microplate reader (Multiskan Go, F1-01620, Thermo Fisher Scientific, Vantaa, Finland). All tests were performed in triplicate. The antioxidant activity was expressed as follows:

% inhibition = 100 × (A _blank_ − A _sample_)/A _blank_,

The A _blank_ is the absorbance of the solvent and DPPH• radical without samples.

### 3.8. Biological Activities

#### 3.8.1. Anti-Inflammatory Activity (15-LOX)

The 15-lipoxygenase enzyme’s ability to oxidize linoleic acid into conjugated diene was measured spectrophotometrically to determine the seed extract’s anti-inflammatory efficacy, as described by Ben Khadher et al. [58]. Briefly, in a 96-well microplate, 20 µL of each extract at a concentration of 625 mg/L were mixed with 150 μL of phosphate buffer (0.1 M, pH 7.4), 60 μL of linoleic acid, and 20 µL of 5-LOX enzyme solution. Following a 10 min incubation period at 25 °C, the mixture’s absorbance was measured at 234 nm. Nordihydroguaiaretic acid (NDGA) at a concentration of 4 mg/L was used as a positive control. The anti-inflammatory activity was expressed as the percentage of inhibition of the 15-LOX enzyme, calculated as:

% inhibition = 100 × (A _blank_ − A _sample_)/A _blank_.

#### 3.8.2. Cytotoxicity Evaluation

##### Cell Culture Growth and Maintenance

The anti-proliferative activity of different rutabaga seed extracts was estimated on human colon adenocarcinoma cell lines (Caco-2), and their cytotoxicity was also evaluated in human embryonic kidney cells (HEK-293) purchased both from the American Type Culture Collection (ATCC, Manassas, VA, USA). Cells were grown in DMEM high glucose (Dulbecco’s Modified Eagle’s Medium, France) supplemented with 10% decomplemented fetal bovine serum, 1% non-essential amino acids, and antibiotics such as penicillin, streptomycin, and gentamicin. Cells were nurtured at 37 °C in a humidified incubator in the presence of 5% carbon dioxide (CO_2_). At 70–80% confluence, cells were harvested and used to perform the cytotoxicity tests.

Adherent cells were seeded in a 96-well microplate at a density of 12 × 10^3^ cells/well for Caco-2 and HEK 293 and were then incubated overnight at 37 °C in a fully humidified atmosphere enriched with 5% CO_2_. Afterwards, cells were treated with each diluted extract at different concentrations (50 µg/mL and 5 µg/mL) in triplicate and re-incubated for an additional 48 h at 37 °C.

##### MTT Assay

The cytotoxicity of extracts was evaluated by the 3-(4, 5-dimethylthiazol-2-yl)-2,5-diphenyltetrazolium bromide (MTT) assay as described by Ben Khadher et al. [58] with slight modifications. Briefly, after 48 h of incubation, the medium was removed and replaced with 50 µL of 1 mg/mL MTT (Sigma-Aldrich) solution, and then the plate was incubated for another 40 min. The dark-blue formazan crystals, produced via the reduction of yellow soluble MTT by mitochondrial dehydrogenase enzymes in viable cells, were dissolved in 80 µL of DMSO, and the absorbance was measured spectrophotometrically at 605 nm using a microplate reader (Multiskan Go, F1-01620, Thermo Fisher Scientific, Vantaa, Finland). Tamoxifen at 100 µM was used as an anti-cancer drug reference.

### 3.9. Statistical Analysis

The obtained data were presented as mean values ± standard deviations, with triplicate measurements performed for each sample. Statistical significance was determined using the ANOVA test accompanied by Tukey’s test to assess the significant difference (*p* < 0.05). All statistical analyses were performed using XL-STAT 2009.

## 4. Conclusions

The present investigation provides valuable insights into the phytochemical profile and in vitro biological properties of rutabaga seed extracts. The HPLC-DAD analysis revealed the presence of four phenolic compounds newly identified in rutabaga organs ((±) synephrine, gallic acid, *p*-coumaric acid, and *trans*-ferulic acid), in addition to ten volatile compounds identified through GC-MS analysis (1,1′-bicyclohexyl; phenol, 3,5-bis(1,1-dimethylethyl)-; 2,4-Di-tert-butylphenol; tetracosanoic acid; methyl ester; docosanoic acid; ethanamine; ethylene glycol; carbonic acid; glycerol; and methyl α-D-glucofuranoside). The biological investigation of the different rutabaga seed extracts revealed moderate anti-DPPH activity, with high inhibition in the methanol extract, and low cytotoxic activity against Caco-2 cell lines. Furthermore, based on their specific chemical composition, CYHA, DCM, and EtOAc extracts demonstrated a moderate level of anti-15-LOX activity. The non-cytotoxicity against the HEK-293 cell lines underscores the rutabaga seed’s safety and non-toxicity for possible utilization in diverse domains, including pharmaceuticals, nutraceuticals, and functional foods. To fully comprehend the properties of the unknown peaks, conducting additional studies using advanced analytical techniques such as LC-HRMS (Liquid Chromatography High-Resolution Mass Spectrometry) and 2D NMR (Two-Dimensional Nuclear Magnetic Resonance) is necessary. These advanced analytical methods are essential for gaining a deeper understanding of the nature and composition of these peaks.

## Figures and Tables

**Table 1:** Extraction yield (DW %) and total phenolic content (TPC, mg GAE/g DW) of different rutabaga seed extracts (CYHA: cyclohexane; DCM: dichloromethane; EtOAc: ethyl acetate; MeOH: methanol, and H_2_O: water).

	Fractional Extraction				
	CYHA	DCM	EtOAc	MeOH	H_2_O
Yields (%)	16.80	7.50	3.65	5.30	5.85
Sugars (mg GE/g DW)	nd	nd	327.40 ± 6.98 ^b^	473.75 ± 36.19 ^a^	189.87 ± 30.48 ^c^
TPC (mg GAE/g DW)	3.68 ± 0.65 ^d^	9.46 ± 2.37 ^c^	9.95 ± 1.06 ^c^	43.77 ± 1.48 ^a^	25.23 ± 3.33 ^b^

Note: nd: not detected; GE: glucose equivalent; GAE: gallic acid equivalents; DW: dry weight. Results are mean ± SD (*n* = 3). The presence of distinct letters ^a, b, c, d^ indicates a significant difference between extracts, according to Tukey’s test (*p* ≤ 0.05).

**Table 2:** Identification and quantification of phenolic compounds in rutabaga seed extracts via HPLC-DAD analysis at 280 nm (CYHA: cyclohexane, DCM: dichloromethane, EtOAc: ethyl acetate, MeOH: methanol, water: H_2_O).

N°	Compounds	RT (min)	Calibration Curves	Concentration (mg/g of Extract)					Ref.
				CYHA	DCM	EtOAc	MeOH	H_2_O	
1	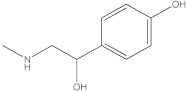 (±) synephrine	1.08	y = 0.385x + 0.5679 R^2^ = 0.9897	0.21	0.27	0.25	-	-	[30]
2	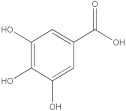 Gallic acid	1.98	y = 0.9282x − 4.35 R^2^ = 0.9939	-	-	-	-	0.46	[27]
3	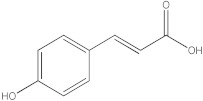 *p*-coumaric acid	3.84	y = 4.8392x + 4.714 R^2^ = 0.9906	-	-	-	5.64	0.66	[28]
4	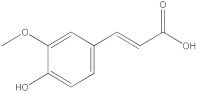 *trans*-ferulic acid	4.26	y = 2.1952x + 14.822 R^2^ = 0.9939	-	1.62	-	-	-	[29]

**Table 3:** Volatile compound profiles of different rutabaga seed extracts using GC-MS before and after derivatization (CYHA: cyclohexane, DCM: dichloromethane, EtOAc: ethyl acetate, MeOH: methanol, water: H_2_O).

N°	RT (min)	Volatile Compounds	Area (×10^7^)					Ref.
			CYHA	DCM	EtOAc	MeOH	H_2_O	
Before Derivatization								
1	14.97	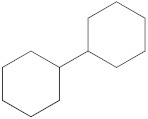 1,1′-Bicyclohexyl	2.55	-	-	-	-	[33]
2	17.87	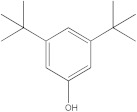 Phenol, 3,5-bis(1,1-dimethylethyl)-	-	4.37	-	-	-	[34]
3	17.87	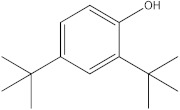 2,4-Di-tert-butylphenol	-	-	1.13	-	-	[31]
4	21.07	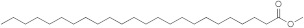 Tetracosanoic acid, methyl ester	8.11	-	-	-	-	[35]
5	21.84	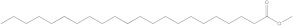 Docosanoic acid, methyl ester	19.47	-	-	-	-	[36]
After Derivatization								
1′	8.28	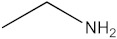 Ethanamine	1.27	-	0.86	-	-	[37]
2′	8.71	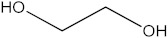 Ethylene glycol	-	0.56	-	-	-	[38]
3′	8.86	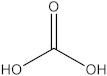 Carbonic acid	-	-	4.44	-	-	[39]
4′	13.19	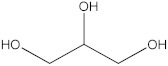 Glycerol	-	0.91	-	-	-	[32]
5′	19.66	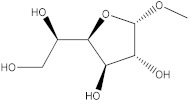 Methyl α-D-glucofuranoside	-	-	-	-	1.51	[40]

**Table 4:** Contribution of variable factors to PCA (%).

	F1	F2	F3	F4
Total phenolic content (TPC)	14.000	0.735	4.401	9.667
Antioxidant activity DPPH	14.369	1.725	1.196	21.841
Anti-15-lipoxygenase activity (15-LOX)	11.785	13.439	0.192	0.474
Cytotoxic activity (HEK-293)	10.513	7.449	8.922	38.434
Cytotoxic activity (HEK-293)	13.220	8.189	0.197	0.260
Cytotoxic activity (Caco-2)	4.576	12.326	28.910	0.184
(±) synephrine	12.948	8.138	0.965	5.458
Gallic acid	1.051	40.469	11.994	16.711
*p*-coumaric acid	12.381	2.735	8.677	6.839
*trans*-ferulic acid	5.157	4.795	34.546	0.133

**Table 5:** Correlations between variables and factors.

	F1	F2	F3
TPC	0.950	0.114	0.273
DPPH	0.963	0.174	0.142
15-LOX	−0.872	−0.486	−0.057
HEK-293	−0.823	0.362	−0.388
HEK-293	−0.923	0.380	0.058
Caco-2	−0.543	0.466	0.699
(±) synephrine	−0.914	−0.378	0.128
Gallic acid	0.260	0.844	−0.450
*p*-coumaric acid	0.893	−0.219	0.383
*trans*-ferulic acid	−0.577	0.291	0.764

TPC: total phenolic content, DPPH: antioxidant activity, 15-LOX: anti-15-lipoxygenase activity, HEK-293 and Caco-2: cytotoxic activity.

**Table 6:** Correlation matrix (Pearson (n)).

Variables	TPC	DPPH	15-LOX	HEK-293	HEK-293	Caco-2	(±) Synephrine	Gallic Acid	*p*-Coumaric Acid	*trans*-Ferulic Acid
TPC	1	0.958	−0.897	−0.867	−0.820	−0.271	−0.869	0.234	0.920	−0.308
DPPH	0.958	1	−0.935	−0.754	−0.812	−0.345	−0.939	0.313	0.889	−0.394
15-LOX	−0.897	−0.935	1	0.559	0.616	0.208	0.975	−0.609	−0.696	0.318
HEK-293	−0.867	−0.754	0.559	1	0.878	0.342	0.550	0.239	−0.946	0.286
HEK-293	−0.820	−0.812	0.616	0.878	1	0.718	0.706	0.052	−0.885	0.687
Caco-2	−0.271	−0.345	0.208	0.342	0.718	1	0.410	−0.061	−0.321	0.982
(±) synephrine	−0.869	−0.939	0.975	0.550	0.706	0.410	1	−0.605	−0.691	0.513
Gallic acid	0.234	0.313	−0.609	0.239	0.052	−0.061	−0.605	1	−0.136	−0.250
*p*-coumaric acid	0.920	0.889	−0.696	−0.946	−0.885	−0.321	−0.691	−0.136	1	−0.286
*trans*-ferulic acid	−0.308	−0.394	0.318	0.286	0.687	0.982	0.513	−0.250	−0.286	1

TPC: total phenolic content, DPPH: antioxidant activity, 15-LOX: anti-15-lipoxygenase activity, HEK-293 and Caco-2: cytotoxic activity.

## Data Availability

The study did not report any data.

## Supplementary Materials

The following supporting information can be downloaded at: https://www.mdpi.com/article/10.3390/molecules28176250/s1, Figure S1: Anti-inflammatory activity (15-LOX) of rutabaga extracts tested at 50 µg/mL NDGA (4 µg/mL). Results are means ± SD (*n* = 3). The histograms’ different letters indicate significant difference according to Tukey’s test (*p* ≤ 0.05); Figure S2: Cytotoxic activity of rutabaga extracts tested at 50 µg/mL against Caco-2 and Tamoxifen (100 µM). Results are means ± SD (*n* = 3). The histograms’ different letters indicate significant difference according to Tukey’s test (*p* ≤ 0.05).

## References

[1] Chopra B., Dhingra A.K. (2021). Natural Products: A Lead for Drug Discovery and Development. Phytother. Res..

[2] Dehelean C.A., Marcovici I., Soica C., Mioc M., Coricovac D., Iurciuc S., Cretu O.M., Pinzaru I. (2021). Plant-Derived Anticancer Compounds as New Perspectives in Drug Discovery and Alternative Therapy. Molecules.

[3] Salehi B., Quispe C., Butnariu M., Sarac I., Marmouzi I., Kamle M., Tripathi V., Kumar P., Bouyahya A., Capanoglu E. (2021). Phytotherapy and Food Applications from Brassica Genus. Phytother. Res..

[4] Ayadi J., Debouba M., Rahmani R., Bouajila J. (2022). Brassica Genus Seeds: A Review on Phytochemical Screening and Pharmacological Properties. Molecules.

[5] Favela-González K.M., Hernández-Almanza A.Y., De la Fuente-Salcido N.M. (2020). The Value of Bioactive Compounds of Cruciferous Vegetables (*Brassica*) as Antimicrobials and Antioxidants: A Review. J. Food Biochem..

[6] Mattosinhos P.d.S., Sarandy M.M., Novaes R.D., Esposito D., Gonçalves R.V. (2022). Anti-Inflammatory, Antioxidant, and Skin Regenerative Potential of Secondary Metabolites from Plants of the Brassicaceae Family: A Systematic Review of In Vitro and In Vivo Preclinical Evidence (Biological Activities Brassicaceae Skin Diseases). Antioxidants.

[7] Le T.N., Sakulsataporn N., Chiu C.-H., Hsieh P.-C. (2020). Polyphenolic Profile and Varied Bioactivities of Processed Taiwanese Grown Broccoli: A Comparative Study of Edible and Non-Edible Parts. Pharmaceuticals.

[8] Peña M., Guzmán A., Martínez R., Mesas C., Prados J., Porres J.M., Melguizo C. (2022). Preventive Effects of Brassicaceae Family for Colon Cancer Prevention: A Focus on in Vitro Studies. Biomed. Pharmacother..

[9] Chalhoub B., Denoeud F., Liu S., Parkin I.A.P., Tang H., Wang X., Chiquet J., Belcram H., Tong C., Samans B. (2014). Early Allopolyploid Evolution in the Post-Neolithic *Brassica napus* Oilseed Genome. Science.

[10] Nawaz H., Shad M.A., Muzaffar S. (2018). Phytochemical Composition and Antioxidant Potential of Brassica. Brassica Germplasm—Characterization, Breeding and Utilization.

[11] Pasko P., Bukowska-Strakova K., Gdula-Argasinska J., Tyszka-Czochara M. (2013). Rutabaga (*Brassica napus* L. Var. Napobrassica) Seeds, Roots, and Sprouts: A Novel Kind of Food with Antioxidant Properties and Proapoptotic Potential in Hep G2 Hepatoma Cell Line. J. Med. Food.

[12] Lim T.K., Lim T.K. (2015). Brassica napus Var. Napobrassica. Edible Medicinal and Non Medicinal Plants: Volume 9, Modified Stems, Roots, Bulbs.

[13] Lugast A., Hovari J. (2000). Flavonoid Aglycons in Foods of Plant Origin I. Vegetables. Acta Aliment..

[14] Carlson D.G., Daxenbichler M.E., VanEtten C.H., Tookey H.L., Williams P.H. (1981). Glucosinolates in Crucifer Vegetables: Turnips and Rutabagas. J. Agric. Food Chem..

[15] Velasco P., Soengas P., Vilar M., Cartea M.E., del Rio M. (2008). Comparison of Glucosinolate Profiles in Leaf and Seed Tissues of Different *Brassica napus* Crops. J. Am. Soc. Hortic. Sci..

[16] Mullin W., COLLINS M., Proudfoot K. (1980). Glucosinolate Content and Clubroot of Rutabaga and Turnip. Can. J. Plant Sci..

[17] Raboanatahiry N., Li H., Yu L., Li M. (2021). Rapeseed (*Brassica napus*): Processing, Utilization, and Genetic Improvement. Agronomy.

[18] Danlami U., Orishadipe Abayomi T., Lawal D.R. (2016). Phytochemical, Nutritional and Antimicrobial Evaluations of the Aqueous Extract of *Brassica nigra* (*Brassicaceae*) Seeds. Am. J. Appl. Chem..

[19] Grygier A. (2022). Mustard Seeds as a Bioactive Component of Food. Food Rev. Int..

[20] Stefanucci A., Zengin G., Llorent-Martinez E.J., Dimmito M.P., Della Valle A., Pieretti S., Ak G., Sinan K.I., Mollica A. (2020). Chemical Characterization, Antioxidant Properties and Enzyme Inhibition of Rutabaga Root’s Pulp and Peel (*Brassica napus* L.). Arab. J. Chem..

[21] Lv X., Meng G., Li W., Fan D., Wang X., Espinoza-Pinochet C.A., Cespedes-Acuña C.L. (2020). Sulforaphane and Its Antioxidative Effects in Broccoli Seeds and Sprouts of Different Cultivars. Food Chem..

[22] Krishnaveni M., Saranya S. (2016). Secondary metabolites, antioxidant activity, phytonutrient analysis of *Nigella sativa* and *Brassica hirta* seeds. Int. J. Pharm. Biol. Sci..

[23] Ogidi O.I., Omu O., Ezeagba P.A. (2019). Ethno Pharmacologically Active Components of *Brassica juncea* (Brown Mustard) Seeds. Int. J. Pharm. Res. Dev..

[24] Jun H.-I., Wiesenborn D.P., Kim Y.-S. (2014). Antioxidant Activity of Phenolic Compounds from Canola (*Brassica napus*) Seed. Food Sci. Biotechnol..

[25] Oh S., Kim K., Choi M. (2016). Antioxidant Activity of Different Parts of Dolsan Leaf Mustard. Food Sci. Biotechnol..

[26] Paśko P., Galanty A., Żmudzki P., Gdula-Argasińska J., Zagrodzki P. (2019). Influence of Different Light Conditions and Time of Sprouting on Harmful and Beneficial Aspects of Rutabaga Sprouts in Comparison to Their Roots and Seeds. J. Sci. Food Agric..

[27] Testai L., Pagnotta E., Piragine E., Flori L., Citi V., Martelli A., Mannelli L.D.C., Ghelardini C., Matteo R., Suriano S. (2022). Cardiovascular Benefits of Eruca Sativa Mill. Defatted Seed Meal Extract: Potential Role of Hydrogen Sulfide. Phytother. Res..

[28] Nicácio A.E., Rodrigues C.A., Visentainer J.V., Maldaner L. (2021). Evaluation of the QuEChERS Method for the Determination of Phenolic Compounds in Yellow (*Brassica alba*), Brown (*Brassica juncea*), and Black (*Brassica nigra*) Mustard Seeds. Food Chem..

[29] Oniszczuk A., Olech M. (2016). Optimization of Ultrasound-Assisted Extraction and LC-ESI–MS/MS Analysis of Phenolic Acids from *Brassica oleracea* L. Var. Sabellica. Ind. Crops Prod..

[30] Zheng G., Yang X., Chen B., Chao Y., Hu P., Cai Y., Wu B., Wei M. (2020). Identification and Determination of Chemical Constituents of Citrus Reticulata Semen through Ultra High Performance Liquid Chromatography Combined with Q Exactive Orbitrap Tandem Mass Spectrometry. J. Sep. Sci..

[31] Hamed M.A., Aboul Naser A.F., Aboutabl M.E., Osman A.F., Hassan E.E., Aziz W.M., Khalil W.K., Farghaly A.A., El-Hagrassi A.M. (2021). Bioactive Compounds and Therapeutic Role of *Brassica oleracea* L. Seeds in Rheumatoid Arthritis Rats via Regulating Inflammatory Signalling Pathways and Antagonizing Interleukin-1 Receptor Action. Biomarkers.

[32] Baky M.H., Shamma S.N., Xiao J., Farag M.A. (2022). Comparative Aroma and Nutrients Profiling in Six Edible versus Nonedible Cruciferous Vegetables Using MS Based Metabolomics. Food Chem..

[33] Kafaltiya M., Lohani H., Bhandari U., Zafar Haider S., Chauhan N., Ahuja T.M., Pant S., Joshi N. (2022). Chemical Composition, Antioxidant and Antimicrobial Potential of the Essential Oils from Aerial Parts of *Tagetes patula* L. at Different Phenological Stages. J. Essent. Oil Bear. Plants.

[34] Selvaraju R., Sakuntala P., Jaleeli K. (2021). GC–MS and FTIR Analysis of Chemical Compounds in *Ocimum gratissimum* Plant. Biophysics.

[35] Shilpa K., Sangeetha K.N., Muthusamy V.S., Sujatha S., Lakshmi B.S. (2009). Probing Key Targets in Insulin Signaling and Adipogenesis Using a Methanolic Extract of Costus Pictus and Its Bioactive Molecule, Methyl Tetracosanoate. Biotechnol. Lett..

[36] Amado P.A., Ferraz V., da Silva D.B., Carollo C.A., Castro A.H.F., Alves Rodrigues dos Santos lima L. (2018). Chemical Composition, Antioxidant and Cytotoxic Activities of Extracts from the Leaves of Smilax Brasiliensis Sprengel (Smilacaceae). Nat. Prod. Res..

[37] Cheng S., Fu X., Wang X., Liao Y., Zeng L., Dong F., Yang Z. (2017). Studies on the Biochemical Formation Pathway of the Amino Acid L-Theanine in Tea (*Camellia sinensis*) and Other Plants. J. Agric. Food Chem..

[38] Salusjärvi L., Havukainen S., Koivistoinen O., Toivari M. (2019). Biotechnological Production of Glycolic Acid and Ethylene Glycol: Current State and Perspectives. Appl. Microbiol. Biotechnol..

[39] Lee J.H., Choi E.J., Chang J.Y., Song K.B., Chun H.H. (2021). Effect of High Hydrostatic Pressure (HHP) and Supercooling Storage in Leaf Mustard (*Brassica juncea* L.) Kimchi: Modelling of Microbial Activity and Preservation of Physicochemical Properties. LWT.

[40] Hassan M., El-Badawy S., Draz M., Ibrahim E. (2021). New Acaricidal Activities and Chemical Compositions of Orange Oil and Extracts of (Wild Mint and Henna) against Tetranychus Urticae Koch (Acari.: Tetranychidae). Arch. Phytopathol. Plant Prot..

[41] Liu X., Ji D., Cui X., Zhang Z., Li B., Xu Y., Chen T., Tian S. (2020). P-Coumaric Acid Induces Antioxidant Capacity and Defense Responses of Sweet Cherry Fruit to Fungal Pathogens. Postharvest Biol. Technol..

[42] Gao J., Hu J., Hu D., Yang X. (2019). A Role of Gallic Acid in Oxidative Damage Diseases: A Comprehensive Review. Nat. Prod. Commun..

[43] Šamec D., Salopek-Sondi B., Nabavi S.M., Silva A.S. (2019). Cruciferous (Brassicaceae) Vegetables. Nonvitamin and Nonmineral Nutritional Supplements.

[44] Parikh H., Khanna A. (2014). Pharmacognosy and Phytochemical Analysis of *Brassica juncea* Seeds. Pharmacogn. J..

[45] Aziz S.S., El-Zayat M.M., El-Khateeb A.Y. (2020). Phytochemical Characterization, Antioxidant and Antimicrobial Activities of *Brassica juncea* (L.) Mustard Seeds Aqueous and Ethanolic Extracts. J. Plant Prod..

[46] Rahmani R., Bouajila J., Jouaidi M., Debouba M. (2020). African Mustard (*Brassica tournefortii*) as Source of Nutrients and Nutraceuticals Properties. J. Food Sci..

[47] Hassanein E.H., Abdel-Wahab B.A., Ali F.E., Abd El-Ghafar O.A., Kozman M.R., Sharkawi S.M. (2021). Trans-Ferulic Acid Ameliorates Cisplatin-Induced Testicular Damage via Suppression of TLR4, P38-MAPK, and ERK1/2 Signaling Pathways. Environ. Sci. Pollut. Res..

[48] Nair R.V., Jayasree D.V., Biju P.G., Baby S. (2020). Anti-Inflammatory and Anticancer Activities of Erythrodiol-3-Acetate and 2, 4-Di-Tert-Butylphenol Isolated from *Humboldtia unijuga*. Nat. Prod. Res..

[49] Ruiz J., Pérez C., Pouplana R. (2003). QSAR Study of Dual Cyclooxygenase and 5-Lipoxygenase Inhibitors 2, 6-Di-Tert-Butylphenol Derivatives. Bioorg. Med. Chem..

[50] Ahmad T.B., Rudd D., Kotiw M., Liu L., Benkendorff K. (2019). Correlation between Fatty Acid Profile and Anti-Inflammatory Activity in Common Australian Seafood by-Products. Mar. Drugs.

[51] Miceli N., Cavò E., Ragusa M., Cacciola F., Mondello L., Dugo L., Acquaviva R., Malfa G.A., Marino A., D’Arrigo M. (2020). Brassica Incana Ten. (Brassicaceae): Phenolic Constituents, Antioxidant and Cytotoxic Properties of the Leaf and Flowering Top Extracts. Molecules.

[52] Rizvi S.M.D., Shakil S., Zeeshan M., Khan M.S., Shaikh S., Biswas D., Ahmad A., Kamal M.A. (2014). An Enzoinformatics Study Targeting Polo-like Kinases-1 Enzyme: Comparative Assessment of Anticancer Potential of Compounds Isolated from Leaves of Ageratum Houstonianum. Pharmacogn. Mag..

[53] Wiebe J., Dinsdale C. (1991). Inhibition of Cell Proliferation by Glycerol. Life Sci..

[54] Shaw G., Morse S., Ararat M., Graham F.L. (2002). Preferential Transformation of Human Neuronal Cells by Human Adenoviruses and the Origin of HEK 293 Cells. FASEB J..

[55] Pulix M., Lukashchuk V., Smith D.C., Dickson A.J. (2021). Molecular Characterization of HEK293 Cells as Emerging Versatile Cell Factories. Curr. Opin. Biotechnol..

[56] Malfa G.A., De Leo M., Tundis R., Braca A., Loizzo M.R., Di Giacomo C., Raimondo F.M., Bucchini A.E.A., Acquaviva R. (2022). Biological Investigation and Chemical Study of *Brassica villosa* subsp. Drepanensis (Brassicaeae) Leaves. Molecules.

[57] Kohoude M.J., Gbaguidi F., Agbani P., Ayedoun M.-A., Cazaux S., Bouajila J. (2017). Chemical Composition and Biological Activities of Extracts and Essential Oil of Boswellia Dalzielii Leaves. Pharm. Biol..

[58] Ben Khadher T., Aydi S., Mars M., Bouajila J. (2022). Study on the Chemical Composition and the Biological Activities of Vitis Vinifera Stem Extracts. Molecules.

