# Supplementary material for: The Phytochemical Screening and Biological Properties of Brassica napus L. var. napobrassica (Rutabaga) Seeds
Source: Molecules. 2023 Aug 25;28(17):6250. doi: 10.3390/molecules28176250 (PMC10488400; doi:10.3390/molecules28176250)
Supplement: Supplementary file 1 [file molecules-28-06250-s001.zip › molecules-2505549-supplementary.pdf]

molecules

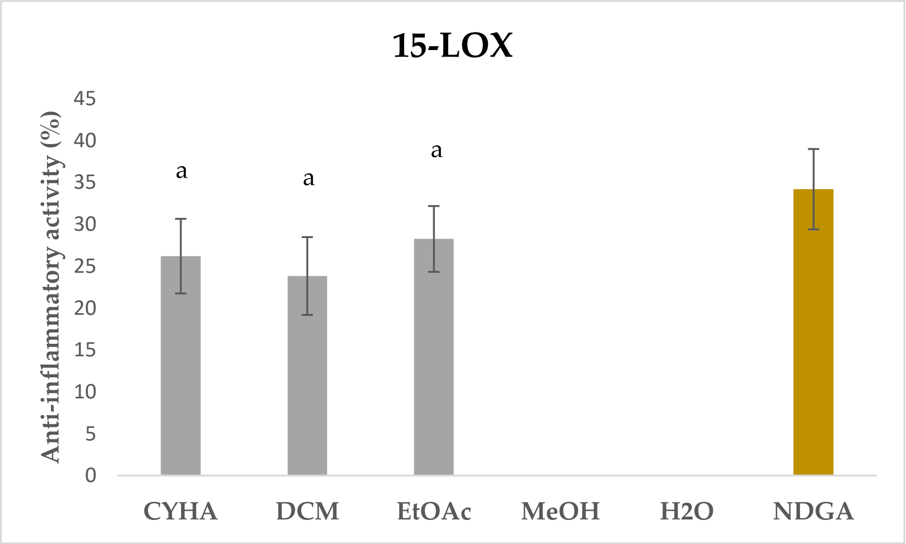

Figure S1. Anti-inflammatory activity (15-LOX) of rutabaga extracts tested at 50 µg/mL NDGA (4 µg/mL). Results are means ± SD (n=3). The histograms' different letters indicate significant difference according to Tukey's test ($p \leq 0.05$).

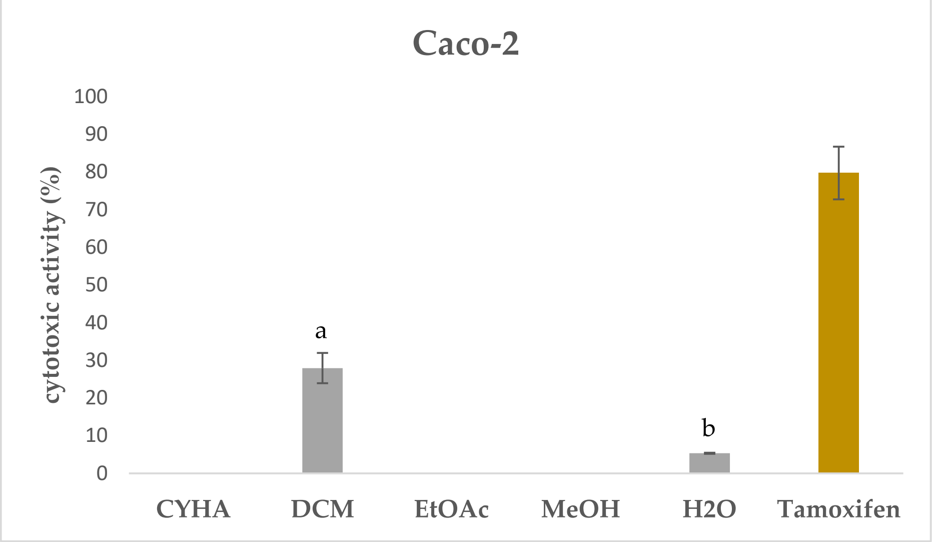

Figure S2. Cytotoxic activity of rutabaga extracts tested at 50 µg/mL against Caco-2 and Tamoxifen (100 µM). Results are means ± SD (n=3). The histograms' different letters indicate significant difference according to Tukey's test ($p \leq 0.05$).
